# Continuous Needle Tip Injection Pressure During Hydrodissection in Four Fascial Plane Blocks: A Fresh Human Cadaver Study

**DOI:** 10.3390/jcm15186979

**Published:** 2026-09-09

**Authors:** Mateusz Wilk, Karol Jędrasiak, Aleksandra Suwalska, Grzegorz Bajor, Jacek Jurkojć, Piotr Wodarski

**Affiliations:** 1 Collegium Medicum, WSB University, 41-300 Dabrowa Gornicza, Poland; 2 Department of Transport and Computer Science, WSB University, 41-300 Dabrowa Gornicza, Poland; 3 Department of Data Science and Engineering, Faculty of Automatic Control, Electronics and Computer Science, Silesian University of Technology, 44-100 Gliwice, Poland; 4 Department of Anatomy, Faculty of Medical Sciences in Katowice, Medical University of Silesia, 40-055 Katowice, Poland; 5 Department of Biomechatronics, Faculty of Biomedical Engineering, Silesian University of Technology, 44-100 Gliwice, Poland

**Keywords:** fascial plane block, injection pressure, hydrodissection, regional anesthesia, pressure monitoring, fresh cadaver

## Abstract

**Background/Objectives**: Continuous injection-pressure monitoring may provide quantitative insight into fascial plane hydrodissection, but comparative data remain limited. This study compared baseline-corrected mean pressure and linear pressure trends during PECS I block (interpectoral plane block) versus superficial parasternal intercostal plane (SPIP) block and rectus sheath block (RSB) versus transversus abdominis plane block (TAPB). **Methods**: Each ultrasound-guided technique was performed once in 10 fresh, unfrozen human cadavers. Saline was infused at 10 mL/min, and pressure was recorded continuously. A deterministic signal-processing algorithm estimated the onset of the post-puncture pressure plateau; the subsequent 40 s segment was analysed after subtraction of the pre-insertion baseline. Paired *t*-tests were used, with exact sign-flip permutation tests as sensitivity analyses and with Holm adjustment for multiplicity. **Results**: Mean pressure was 4.99 psi for PECS I and 5.09 psi for SPIP, with no significant difference between techniques (mean paired difference, 0.09 psi; 95% confidence interval [CI], −0.67 to 0.85; *p* = 0.790). TAPB showed a higher mean pressure than RSB (5.50 versus 4.47 psi, respectively; mean paired difference, 1.03 psi; 95% CI, 0.36 to 1.69; *p* = 0.0134; paired effect size, d = 1.11). Permutation analyses were concordant. Linear pressure trends did not differ for SPIP versus PECS I (*p* = 0.484) or TAPB versus RSB (*p* = 0.742). **Conclusions**: Continuous pressure monitoring was used to characterize the hydrodissection pressure of four fascial plane block techniques. PECS I and SPIP yielded closely aligned mean values, whereas TAPB was associated with higher pressure than RSB. These findings provide quantitative reference data and support further clinical evaluation of continuous injection-pressure monitoring.

## 1. Introduction

Fascial plane blocks are a type of regional anesthetic block in which a local anesthetic is injected into the space between two layers of muscle fascia so that the local anesthetic solution covers the sometimes extensive areas of the interfascial planes where nerves are located [1]. Previous studies have reported that initial injection pressures above 15 PSI—where PSI stands for pounds per square inch, which corresponds to 775.723 mmHg—suggest intraneural injection of the local anesthetic or contact between the needle tip and the fascial layer [2,3,4,5]. In 2016, a study by Gadsden J.C. [6] specifically demonstrated that contact between the needle tip and the fascia causes an initial injection pressure > 15 PSI (=103.42 kPa). Quadri C. [7] followed up on this study by proposing the first system for continuous measurement of injection pressure based on a pressure-sensitive optical sensor located inside the needle body just behind the needle tip. This system was later evaluated and used in subsequent studies, which demonstrated significant differences between the pressure at the very tip of the needle and that inside the needle shaft and tubing, emphasizing the need to monitor pressure at the needle tip [8,9,10,11]. Subsequently, we conducted a study [12] that proposed a much simpler yet reliable method for both locating the interfascial space and measuring pressures during needle penetration (within the muscle, upon penetration into the fascia, and during the opening of the interfascial space), which was also based on measuring pressure at the needle tip, but in relation to the pressure plateau measured prior to tissue puncture.

Compartment blocks differ not only in their anatomical location but also in the depth of needle insertion into the tissues. Some—such as PECS I (also known as interpectoral plane block), the superficial parasternal intercostal block (SPIP), and the Rectus Plane Block (RSB)—require the needle to pass through only one muscle layer to reach the intended fascial space [13,14,15]. Other blocks, such as the transversus abdominis plane block (TAPB) or blocks from the quadratus lumborum (QLB) group, require the needle to pass through multiple muscles and fasciae before reaching the target [16,17]. In light of previous studies, we investigated in this study whether the hydrodissection pressure for various regional blocks differs depending on the type of block.

The primary objective of this study was to compare baseline-corrected mean injection pressure during the stable phase of hydrodissection in two predefined within-specimen anatomical comparisons: PECS I versus SPIP block and RSB versus TAPB. The comparison between PECS I and SPIP was chosen because the injection site was located beneath the posterior fascia of the pectoralis major muscle; thus, both blocks were at least partially connected by a common fascia through which the needle had to be inserted. In the comparison between RSB and TAPB, however, the needle tip was located in completely different fascial spaces belonging to the abdominal wall musculature. The secondary objective was to compare the linear pressure trend during the analyzed hydrodissection segment.

## 2. Materials and Methods

Ethical approval was obtained from the Bioethics Committee of the Medical University of Silesia (BNW/NWN/0052/KB/117/24) prior to conducting the study. The study was conducted at the Department of Anatomy, Medical University of Silesia, Katowice. Ten randomly selected, fresh (unfrozen) human cadavers were used in the study.

The measuring system described in [12] was used. The device consisted of a pressure sensor, a docking port, a button to start recording, a button to mark “events” during recording, and LEDs to indicate operation. The device was powered by the USB port of the computer to which it was connected. The same USB port was used to send data to the computer. An analog-to-digital converter integrated in the pressure sensor measures changes in the stress of the measuring cell, which are directly proportional to the measured pressure. The measured values are read out by a microcontroller via a serial interface and then converted.

The measuring system comprised a Mindray BeneFusion infusion pump, a 60 mL BD PlasticPack infusion syringe, two 150 cm drains, a three-way stopcock, a measuring device, and a SonoBlock II Facet 22G × 80 mm needle provided by Pajunk. The measuring device was connected to the system via a T-connector, with a syringe in the infusion pump attached to one end and two drains connected to a needle attached to the other end. A Mindray M9 ultrasound machine with a linear probe was used for tissue imaging. During the study, saline was continuously administered using an infusion pump at a rate of 10 mL/min (600 mL/h) to conform to the methodology of previous studies, including our own.

The infusion and measurements were started just before tissue penetration, continued during needle maneuvers within the tissues and the hydrodissection of the interfascial plane, and ended after the needle was removed from the tissues. In the first stage of the study, the fluid pressure generated by the measuring system (with the needle not inserted into the tissue) was checked. Once the pressure curve had stabilized (plateau phase), a needle was inserted into the tissues, directed towards the interfascial plane. Throughout the procedure, saline was continuously administered via an infusion pump, which enabled pressure monitoring as the needle progressed through successive tissue layers. Due to the anticipated high injection pressures, the pump’s pressure alarms were deactivated. During the measurements, the needle was at the same level as the pressure sensor. Since the pressure analyzed was referenced to the plateau pressure obtained just before the needle penetrated (touched) the tissue, pressure changes caused by inserting the needle into the tissue (with the needle positioned at the same level as the pressure sensor) that are presented in this study are physically identical to the pressure measured at the tip of the needle.

A PECS I also known as Interpectoral Plane Block is defined as the performance of a hydrodissection between the pectoralis major and pectoralis minor muscles. In this case, the procedure was performed using the in-plane technique (the linear probe was positioned along the long axis of the pectoralis minor muscle); the needle was inserted from the region of the 4th intercostal space toward the coracoid process of the scapula. When the posterior fascia of the pectoralis major muscle was penetrated and hydrodissection (the “kayak sign”) was achieved, needle advancement was halted, and the needle remained stationary within the hydrodissected fascial space for 60 s.

SPIP block is defined as a hydrodissection of the space between the pectoralis major and the intercostal muscles; however, we utilized the methodology proposed by [13,18]. In this block, the linear probe was placed sagittally in the parasternal line, and the needle was advanced in-plane in a caudo-cranial direction until it encountered the rib, and the pectoralis major muscle was separated from the rib by hydrodissection. After this, needle advancement was halted, and the needle remained stationary within the hydrodissected fascial space for 60 s.

RSB refers to hydrodissection between the rectus abdominis muscle and the posterior layer of its sheath in any of the periumbilical quadrants. In this technique, the linear probe was placed transversely over the rectus abdominis muscle in the left or right upper quadrant, above the navel. The needle was inserted in-plane from lateral to medial until it rested against the posterior layer of the rectus abdominis sheath and hydrodissection was achieved between the belly of the muscle and the posterior layer of its sheath. After this, needle advancement was halted, and the needle remained stationary within the hydrodissected fascial space for 60 s.

TAPB refers to hydrodissection between the fasciae of the internal oblique and transverse abdominis muscles along the mid-axillary line. In this block, the linear probe was placed transversely along the mid-axillary line on the right or left side. The needle was inserted using the in-plane technique from front to back, passing through the muscles until it reached the fascial space between the internal oblique muscle and the transverse abdominal muscle (TAP space). Once hydrodissection was achieved in the TAP space, the needle was held stationary for 60 s.

During the study, the following blocks were performed on every cadaver: PECS I (Figure 1 and Figure 2), modified SPIP (Figure 3 and Figure 4), RSB (Figure 5 and Figure 6) and TAPB (Figure 7 and Figure 8), with one block of each type performed on every cadaver.

Cadavers used in the study were selected completely randomly, as it was randomised according to the order in which the bodies were brought to the Department of Anatomy of the Medical University of Silesia in Katowice, Poland. Tests on each cadaver were performed as soon as they arrived at the Department (within 24 h of death), with the air temperature in the laboratory being 20 degrees Celsius. Tissue temperature at the site of the block was not measured to avoid damaging the muscles and fascia, which could have led to inaccurate results.

After locating the interfascial plane with the needle tip in each block, the interfascial injection pressure was measured for 60 s. In the course of the study, 10 mL of saline was introduced into each interfascial space. Every human cadaver had every regional block performed 1 time, with PECS I block and SPIP block performed on the contralateral sides of the human cadaver. Human cadavers were intact at the time of the procedure.

In this study, an anesthesiologist performed the in-plane puncture of the tissues of the cadavers, directing the needle into the mentioned above interfascial spaces until hydrodissection of the fascias of adjacent muscles was achieved (kayak sign). The operator was blinded to the real-time data collected by the engineer. The operator saw only the image on the ultrasound machine. During the measurements, the moments when they passed through the muscle, rested on the fascia, punctured the fascia, and hydrodissected the interfascial space, as well as when the needle exited the fascial space, were noted in the measurement program. The data collected in this way were exported to Excel files. Statistical analysis was performed.

**Automated detection of characteristic points in pressure–time curves**

Pressure–time recordings were analyzed using a custom deterministic signal-processing algorithm implemented in Python 3.13.7 to identify the D1–D2 segment used for the main analysis. Pressure was analyzed in PSI (pounds per square inch). The time axis was referenced to the first valid timestamp in each recording. Recordings were nominally sampled at approximately 100 Hz. The pressure signal was sequentially smoothed using a centered median filter and an exponentially weighted moving average for temporal-landmark identification. All characteristic points and their corresponding pressure values were determined from the filtered signal.

A low-variability reference interval was identified in the filtered signal and used to support localization of the stable post-puncture pressure phase. Point W represented the algorithmically detected initial sustained pressure rise associated with needle insertion. Point D1 was defined as the algorithmically estimated onset of the stable post-puncture pressure plateau. Preliminary high-pressure events following W were used only to delimit the search for D1. Final P1–P4 labels were auxiliary and were not used in the formal between-technique comparisons. The block-specific expected peak count was used only for final P1–P4 labeling and did not determine the D1–D2 segment. Point D2 was defined exactly 40 s after D1.

We developed the segmentation procedure using the same recordings included in the present study. We established its numerical settings empirically during iterative visual development to provide an operational segmentation of characteristic signal transitions; they were not intended as physiological or clinical thresholds. A medical expert independently reviewed the resulting segmentation. The Supplementary Methods provide the equations, numerical settings, temporal criteria, fallback procedures, and complete P1–P4 detection procedure.

Because D1 determined the start of the 40-s segment used for the primary pressure analysis, we performed a post hoc boundary-sensitivity analysis to assess whether the findings depended on its precise algorithmic location. We repeated the complete primary analysis after shifting the beginning of the 40-s interval by each integer value from −5 to +5 s relative to the automatically identified D1.

**Statistical Analysis**

Two paired anatomical comparisons were predefined. PECS I block was compared with the SPIP block because both techniques involve injection beneath the pectoralis major muscle, but in different anatomical regions. Rectus sheath block (RSB) was compared with transversus abdominis plane block (TAPB) because both are anterior abdominal wall fascial plane techniques, although they target distinct interfascial compartments.

**Figure 9:**
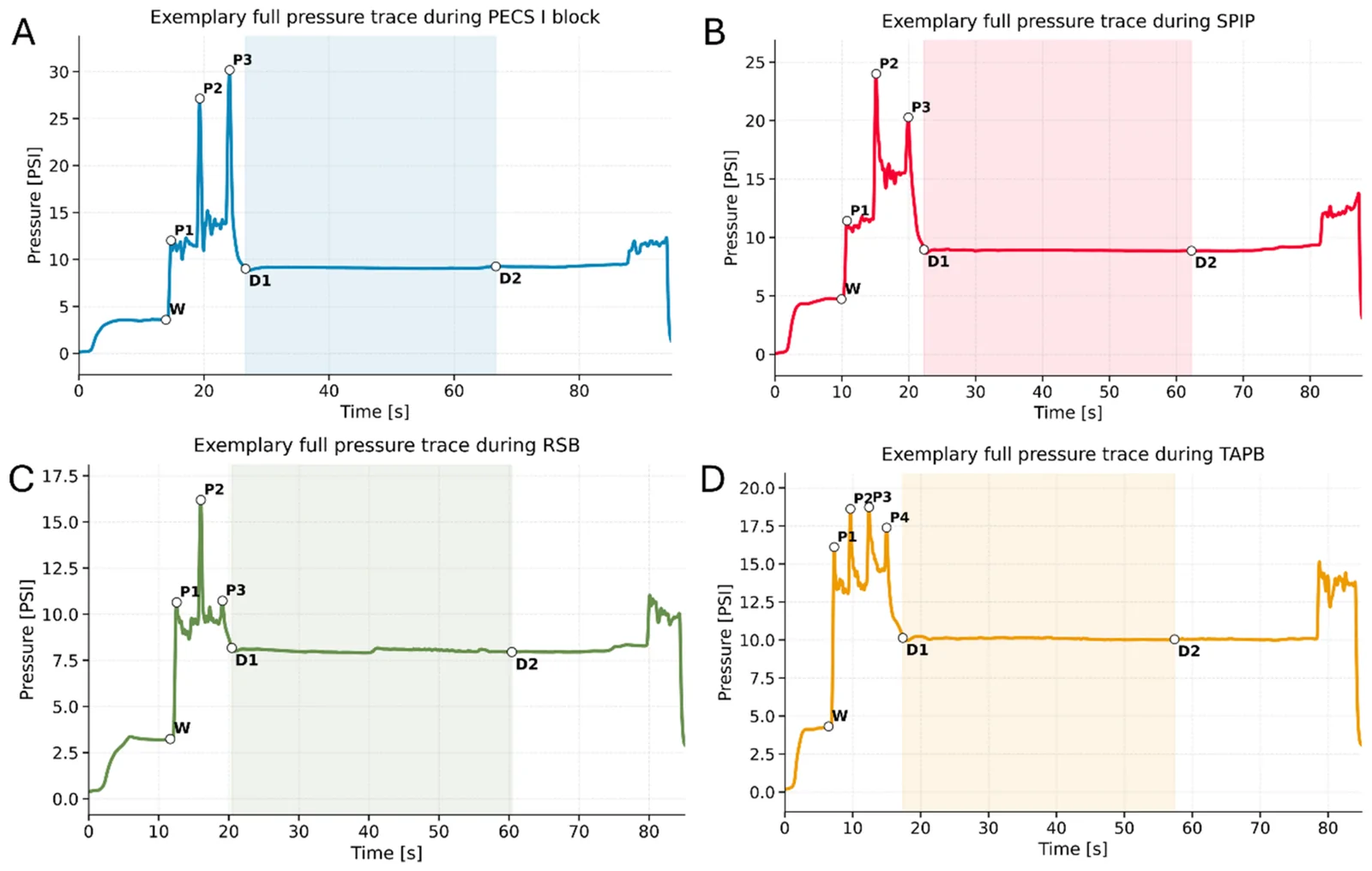
Exemplary pressure traces for each block. (**A**) PECS I Block; (**B**) superficial parasternal intercostal plane block (SPIP Block); (**C**) rectus plane block (RSB); (**D**) transversus abdominis plane block (TAPB). Abbreviations: W—initial sustained pressure rise associated with needle insertion; P1–P4—pressure maxima preceding the stable hydrodissection phase; D1–D2—the stable phase of hydrodissection.

For each pressure trace (Figure 9), the stable phase of hydrodissection (from D1 to D2) within the target fascial plane was identified using an automated signal-processing procedure. Only this stable injection segment (the first 40 s) was used for the main comparative analysis because the system detected increasing pressure fluctuations after this section. Before extracting the pressure parameters, each recording was corrected for baseline pressure. Baseline was defined as the median pressure during the three-second pre-W interval, intended to represent the pre-insertion baseline.

**Figure 10:**
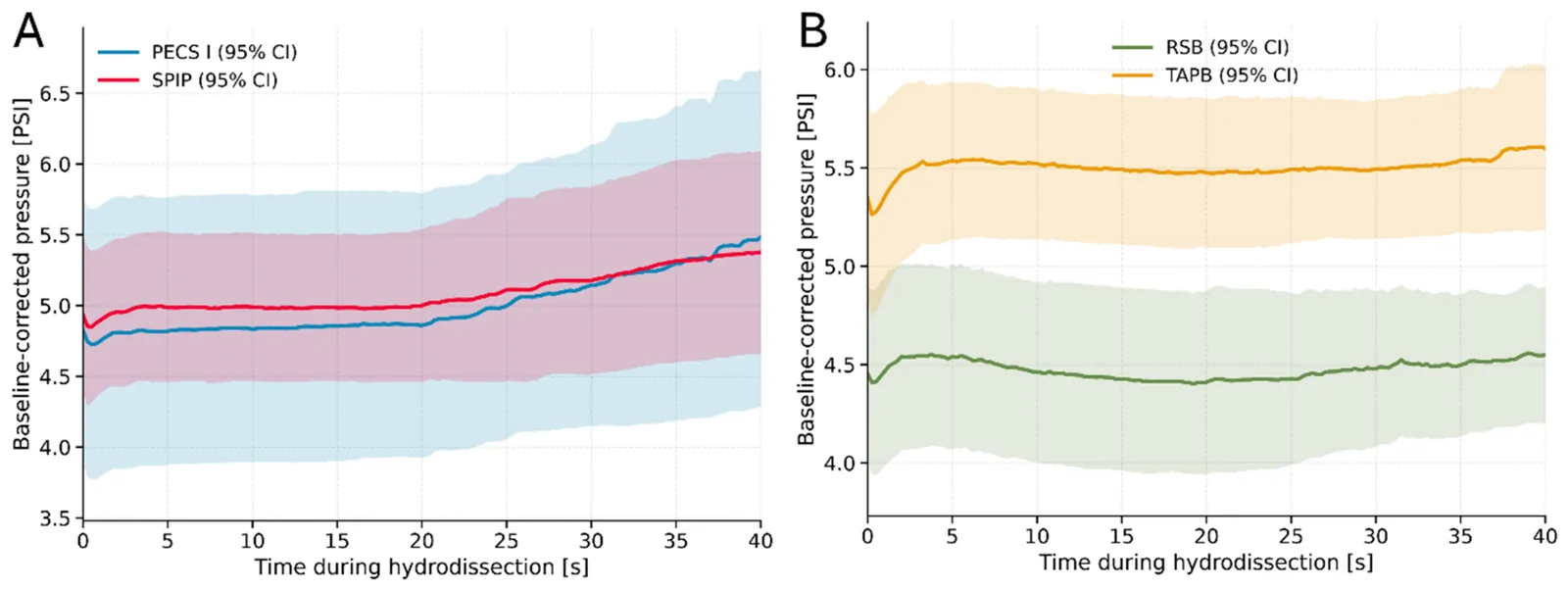
The time-resolved pressure profiles across the stable hydrodissection segment as mean curves with 95% confidence intervals. (**A**) PECS I versus SPIP block; (**B**) RSB versus TAPB.

The time-resolved pressure profiles across the stable hydrodissection segment are presented descriptively in Figure 10 as mean curves with 95% confidence intervals. However, formal inference was based primarily on summary measures derived from each individual recording, especially the mean pressure over the analyzed segment, rather than on point-by-point testing of individual time samples. Pressure peaks detected before the stable hydrodissection phase were summarized descriptively within each block type only and were not used for formal comparisons between block techniques (Supplementary Figure S2 and Table S3).

Because each cadaver underwent all four block procedures, measurements were matched within the same specimen, and each cadaver served as its own experimental unit. Therefore, the predefined anatomical comparisons were analyzed using paired methods.

For each anatomical comparison, paired differences were calculated within the same cadaveric specimen. The distribution of paired differences was assessed using the Shapiro–Wilk test and visual inspection of quantile-quantile plots. As no relevant departures from normality were identified (Supplementary Figure S1), the primary comparative analysis was performed using paired *t*-tests. The two predefined mean-pressure comparisons were treated as one family of primary hypotheses, and the corresponding *p*-values were adjusted using the Holm procedure to control the family-wise type I error rate at 0.05. Analyses of the linear pressure trend were secondary and supportive and were not included in the primary multiplicity adjustment. Paired Cohen’s d effect size was reported as the standardized mean difference for paired data. Because of the small sample size, an exact sign-flip permutation test was additionally performed as a sensitivity analysis for the mean paired difference.

Cadaveric characteristics, including sex, height, body weight, and body mass index, were evaluated exploratorily. These analyses focused on whether specimen characteristics were associated with the paired difference between block techniques, rather than with the absolute pressure values of a single block. For continuous characteristics, correlations with paired pressure differences were assessed. For sex, the distribution of paired differences was compared between female and male specimens. These analyses were considered supportive and hypothesis-generating.

## 3. Results

The analysis included 10 fresh human cadaveric specimens, comprising 5 females and 5 males. The mean height was 167.2 cm, and the mean body weight was 63.3 kg. The mean body mass index was 22.6 kg/m^2^, indicating a relatively homogeneous sample with respect to body habitus (Supplementary Tables S1 and S2). The computed descriptive statistics for the average pressure in the hydrodissection segment are provided in Table 1. Mean injection pressure did not differ significantly between PECS I and SPIP after correction for multiple primary comparisons (Holm-adjusted *p* = 0.790; Table 2).

**Figure 11:**
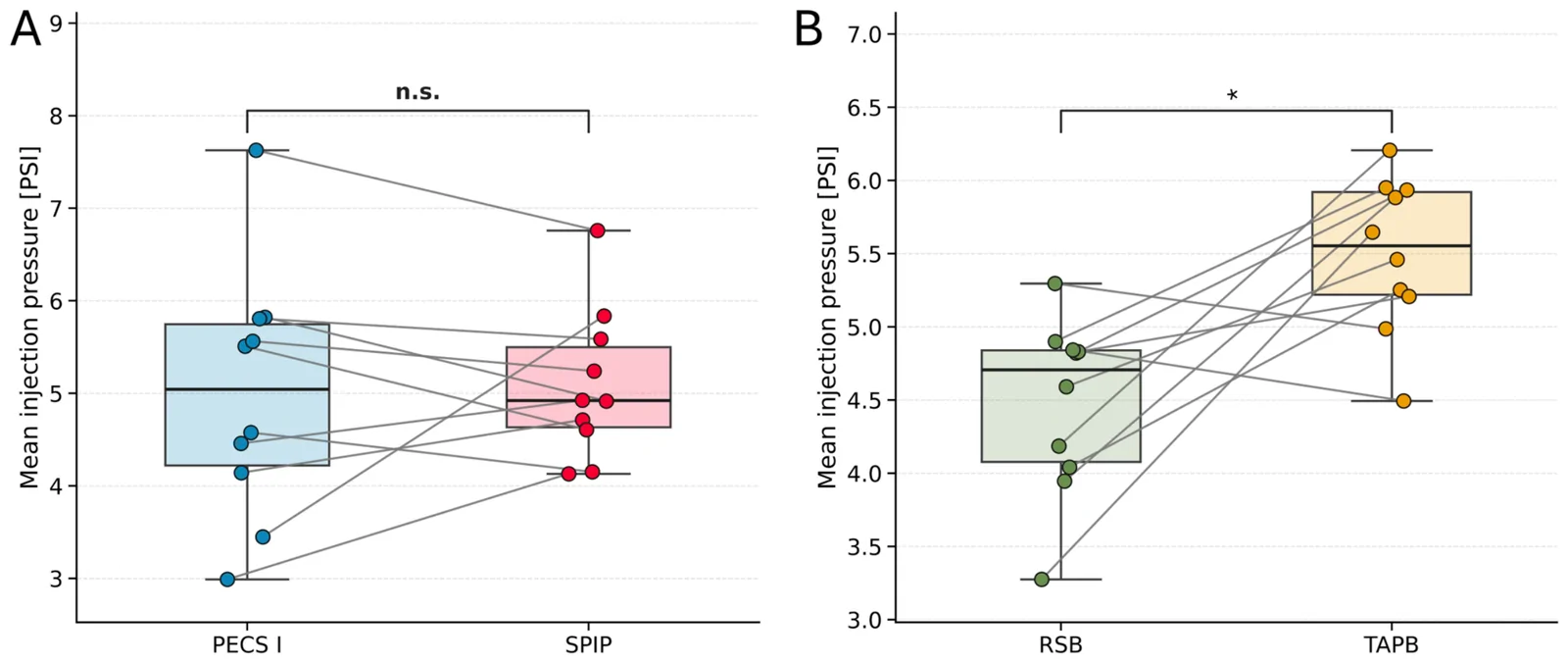
Comparison of the mean injection pressure after baseline correction during the stable hydrodissection phase. Each dot represents an individual mean pressure measurement in one block, connected to the corresponding mean pressure in the other block, showing the change in values. The annotations indicate the *t*-test results: “n.s.”—not significant; “*”—*p*-value below 0.05. (**A**) PECS I block and SPIP block; (**B**) RSB and TAPB.

A different pattern was observed when comparing RSB and TAPB (Figure 11). TAPB showed higher mean injection pressure than RSB, with a mean paired difference of 1.03 PSI (95% CI, 0.36 to 1.69; Holm-adjusted *p* = 0.013; d = 1.11; Table 2).

The analysis of the pressure trend during the hydrodissection phase showed no significant differences between techniques in either anatomical comparison (Table 3). For SPIP block versus PECS I block, the mean paired difference in the linear pressure slope was −0.005 PSI/s, with a 95% confidence interval of −0.021 to 0.011 PSI/s (t = −0.73, *p* = 0.484). For TAPB versus RSB, the mean paired difference in the linear pressure slope was 0.001 PSI/s, with a 95% confidence interval from −0.008 to 0.011 PSI/s (t = 0.34, *p* = 0.742). Thus, the significant difference observed in the second comparison reflected a difference in the overall pressure level rather than a systematic difference in the rate of pressure increase or decrease during the analyzed injection segment.

The sensitivity analysis based on exact sign-flip permutation testing was concordant with the primary parametric analysis (Table 4). For PECS I block versus SPIP block, the difference in mean pressure remained non-significant (Holm-adjusted *p* = 0.809). For RSB versus TAPB, the difference remained statistically significant (*p* = 0.02). Nonparametric paired testing led to the same substantive interpretation and did not alter the conclusions of the primary analysis.

No clear associations were observed in exploratory analyses; however, these analyses were severely underpowered and should not be interpreted as evidence of absence of an association. Height, body weight, and body mass index were evaluated using Pearson correlation with the paired pressure difference (Table 5). Resulting *p*-values were adjusted for multiple exploratory comparisons using the Holm procedure. Sex was evaluated by comparing the distribution of paired pressure differences between female and male specimens using Welch’s *t*-test (PECS I vs. SPIP: *p* = 0.931, RSB vs. TAPB: *p* = 0.996). None of the tested cadaveric covariates showed a statistically significant association with the paired mean-pressure difference after multiplicity correction.

The post hoc D1–D2 segment sensitivity analysis yielded the same substantive interpretation across all tested boundaries (Supplementary Table S4). For SPIP versus PECS I, mean paired differences ranged from 0.047 to 0.198 psi and remained non-significant throughout the D1−5 s to D1+5 s range (Holm-adjusted *p* = 0.603–0.895). For TAPB versus RSB, mean paired differences ranged from 1.022 to 1.087 psi and remained positive and statistically significant throughout the tested range (Holm-adjusted *p* = 0.0096–0.0185). Exact sign-flip permutation analyses were concordant. Thus, the interpretation of both primary comparisons was unchanged throughout the tested D-boundary range.

## 4. Discussion

There are very few articles in the scientific literature dealing with the measurement of interstitial pressure during the performance of regional blocks. In fact, we found only one study, by Roberto D et al. [19], that discussed the injection pressure using a pressure gauge mounted on the tip of the needle. In their study, TAPBs were performed on four cadavers, with measurements taken as the needle passed through the tissues, including the fascia, until hydrodissection of the TAP space was achieved. The results of that study are similar to ours, although we obtained a slightly lower fascial penetration pressure and a slightly higher hydrodissection pressure than those reported by the aforementioned authors. This may have been caused by the type of needle used in the study and the mechanical characteristics of the tissues used in the study. However, the shape of the curve is similar to our findings, as we were able to record four peaks in the case of TAPB, as described below, of which the one additional peak corresponds to the pressure required to penetrate the skin.

Irrespective of the statistical analysis, repeatable patterns of pressure distribution were observed as the probe passed through the skin, subcutaneous tissue and fascia up to the hydrodissection site; their typical shapes are shown in Figure 9. For the PECS I block and the SPIP block, P1 was interpreted as corresponding to penetration of the skin, P2 as penetration of the anterior fascia of the pectoralis major muscle, and P3 as penetration of the posterior fascia of the pectoralis major muscle. For the RSB, P1 denoted penetration of the skin, P2 denoted penetration of the anterior layer of the rectus abdominis sheath, and P3 denoted penetration of the muscle and contact with the posterior layer of the sheath. For TAPB, P1 denoted penetration of the skin, P2 denoted penetration of the anterior fascia of the external oblique muscle, P3 denoted penetration through the fascias of the external and internal oblique muscles, and P4 denoted penetration through the posterior fascia of the internal oblique muscle, followed by entry into the TAP space.

An important aspect of identifying the fascial space in our study was the “kayak sign,” which occurs when the needle tip enters the interfascial space and hydrodissection begins. The differences in saline infusion pressure among the blocks we studied are minor; however, we had previously [12] conducted studies aimed at determining whether there are differences in saline infusion pressure within the muscles, when resting against the fascia and during hydrodissection. In the aforementioned study, we demonstrated that the intramuscular infusion pressure is higher than that during interfascial hydrodissection, which in turn was lower than the pressure when pressing against the fascia. We obtained similar pressure patterns in this study as well, as shown in Figure 9.

An interesting finding is the lack of a difference in injection pressure between SPIP and PECS I. It is a fact that, in both blocks, saline was administered into the fascial space beneath the posterior fascia of the pectoralis major muscle. The lack of a pressure difference possibly indicates that, when saline was administered into a different fascial space—albeit one associated with the same fascial layer—the mechanical properties of the fascial spaces may be similar. A distinct situation was observed in the case of RSB and TAPB, where the pressure of injection generated during TAPB hydrodissection was higher than during RSB hydrodissection. The absolute clinical relevance of the approximately 1 psi difference remains uncertain. This may be influenced by several factors, such as the properties of the fascia, tissue depth, or tissue cohesion and compliance. This issue requires further investigation.

No statistically detectable difference was observed in injection pressure; this finding does not establish mechanical equivalence of the fascial compartments. However, injectate spread is probably multifactorial and may depend on tissue anisotropy, compliance, fascial architecture and transport across tissue boundaries. This is particularly important in the case of local anesthetic solutions, especially bupivacaine. It has been demonstrated that the extent of bupivacaine’s penetration into tissues is much greater than that of crystalloid solutions and water-soluble dye solutions, such as methylene blue. This is because bupivacaine spreads anisotropically within fresh muscle and permeates fascial barriers [19]. Recent cadaveric studies have demonstrated that even closely related thoracic fascial plane approaches may result in substantially different patterns and extent of injectate spread depending on the targeted anatomical plane [20].

The reasons why certain regional blocks may exhibit varying levels of hydrodissection pressure may include differences in the depth at which the block is performed, the cohesiveness and compliance of the muscles surrounding the interfascial space, and the properties of the fascia itself (cohesiveness of the interfascial tissue, stiffness of the fascia) and, interestingly, the shape of the needle tip used to perform the block [21]. These considerations are purely hypothetical and suggest potential directions for further research.

The deterministic segmentation procedure was developed using the pressure recordings included in the present study, and its operational settings were empirically established and independently reviewed by a medical expert. A post hoc boundary-sensitivity analysis showed that the substantive interpretation of both prespecified primary comparisons was unchanged when the D1–D2 segment was displaced by up to ±5 s. This supports the stability of the reported findings with respect to the precise location of the D1–D2. However, independent validation is required before generalizing the method beyond the present analytical setting.

The results of our study may have clinical applications. A continuous injection pressure monitoring system integrated with an operator-controlled infusion pump (such as the Safira pump [22]), with a display showing peaks and drops in injection pressure, could significantly facilitate the performance of regional blocks by tracking the typical pressure patterns presented in this paper and by detecting the delivery pressure of, for example, a local anesthetic in a specific fascial space. This idea is merely a hypothesis and an indication of the direction in which the technology could develop; nevertheless, further extensive research—including both cadaver studies and clinical trials—is necessary for its further development.

A key advantage of this study is the use of fresh, unfrozen cadavers for the experiments, which enabled us to obtain results that would have been impossible to achieve with frozen or Thiel cadavers. We were able to select, entirely at random, a group of cadavers with very similar BMIs, meaning that the variability within the group in terms of conditions was relatively low. Furthermore, as demonstrated above, exploratory analyses did not provide evidence that sex, height, body weight, or body mass index explained the observed differences in pressure between block techniques. Highly sensitive measuring equipment, which had previously been tested on animal tissues, was used for the measurements.

The limitation of our study is that it was not performed in patients, as fascial spread might be influenced by patient position and ventilation. It is also important to note that cadavers lack perfusion, vascular filling, muscle tone, physiological tissue pressure and respiratory movement, all of which may influence fascial compliance and injectate spread. No anatomical dissection or staining assessment was performed. We accept that in clinical practice the density, viscosity, and fluid dynamics of saline solution might be similar but still different from that of local anesthetics.

## 5. Conclusions

Under a standardized saline infusion rate of 10 mL/min in fresh, unfrozen human cadavers, no statistically significant difference in baseline-corrected mean pressure during stable hydrodissection was detected between PECS I and SPIP blocks. In contrast, TAPB was associated with a mean pressure approximately 1.03 psi higher than that of RSB. The findings indicate that pressure conditions during hydrodissection may differ between fascial plane block techniques. However, the present design does not establish whether the observed difference was caused by differences in target depth, fascial anatomy, compartment geometry, or other tissue-specific factors. Clinical validation and studies incorporating direct measurements of block depth are required.

## Figures and Tables

**Figure 1:**
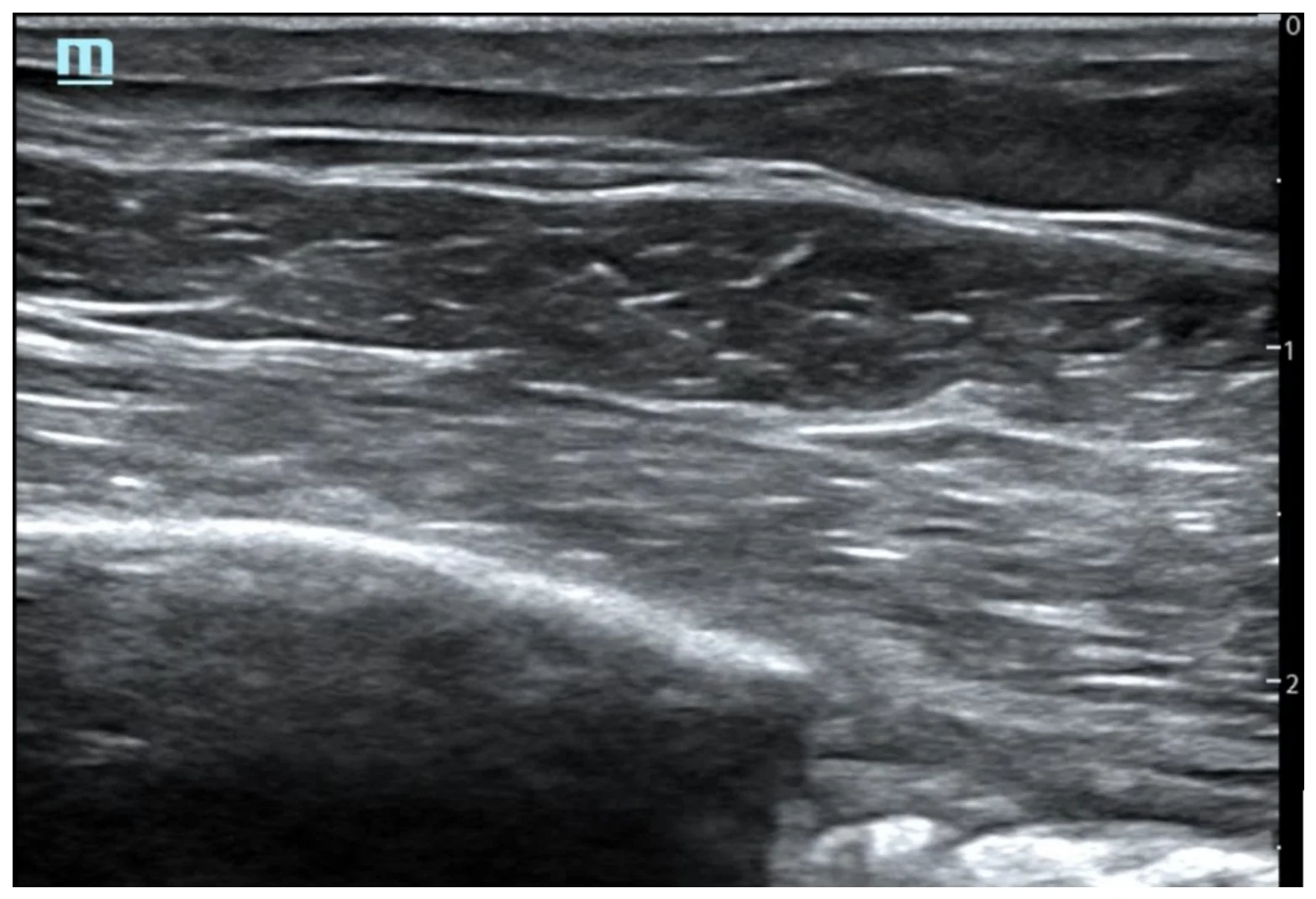
Ultrasound view of pectralis major and minor muscles.

**Figure 2:**
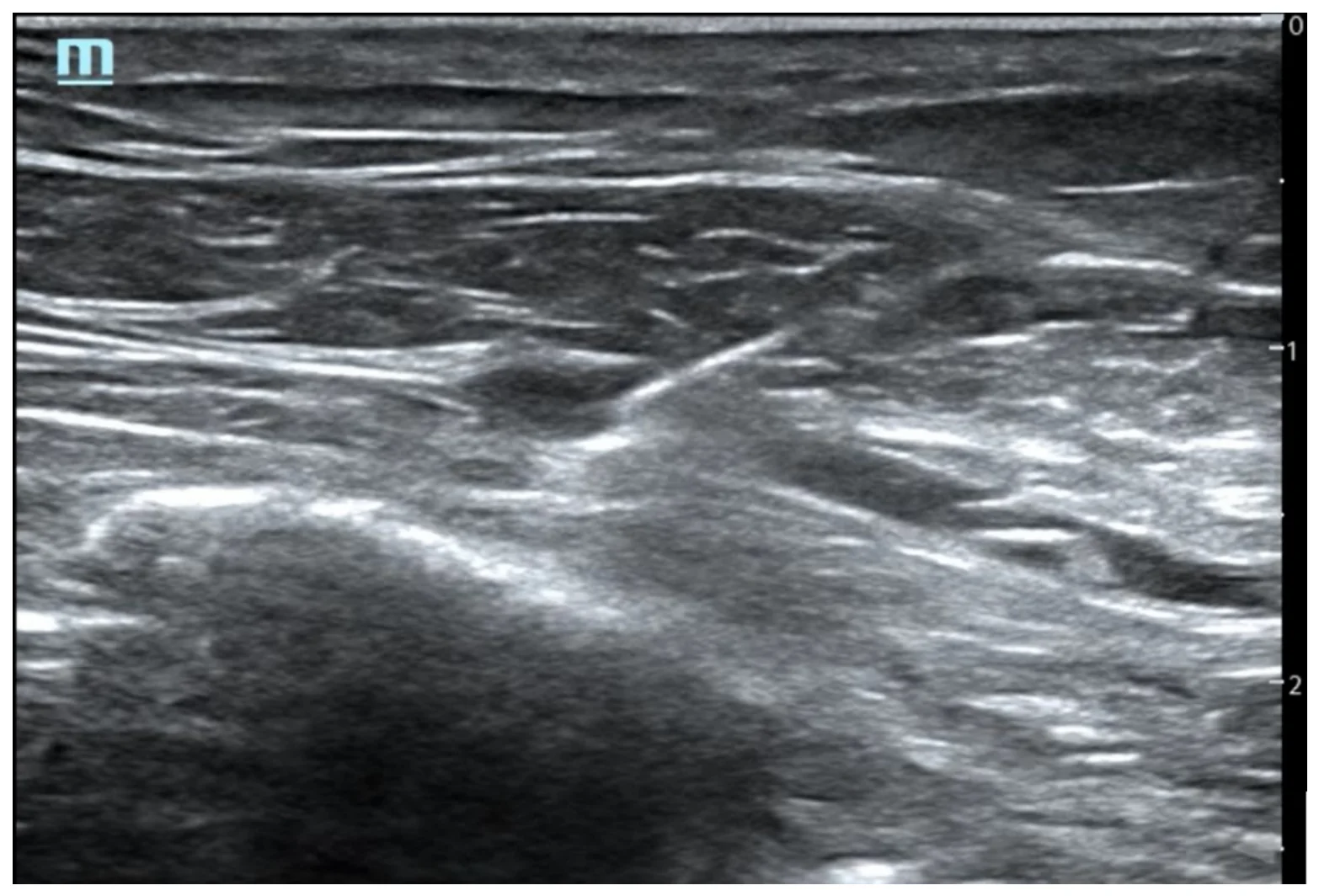
Ultrasound view of hydrodissection during PECS I block.

**Figure 3:**
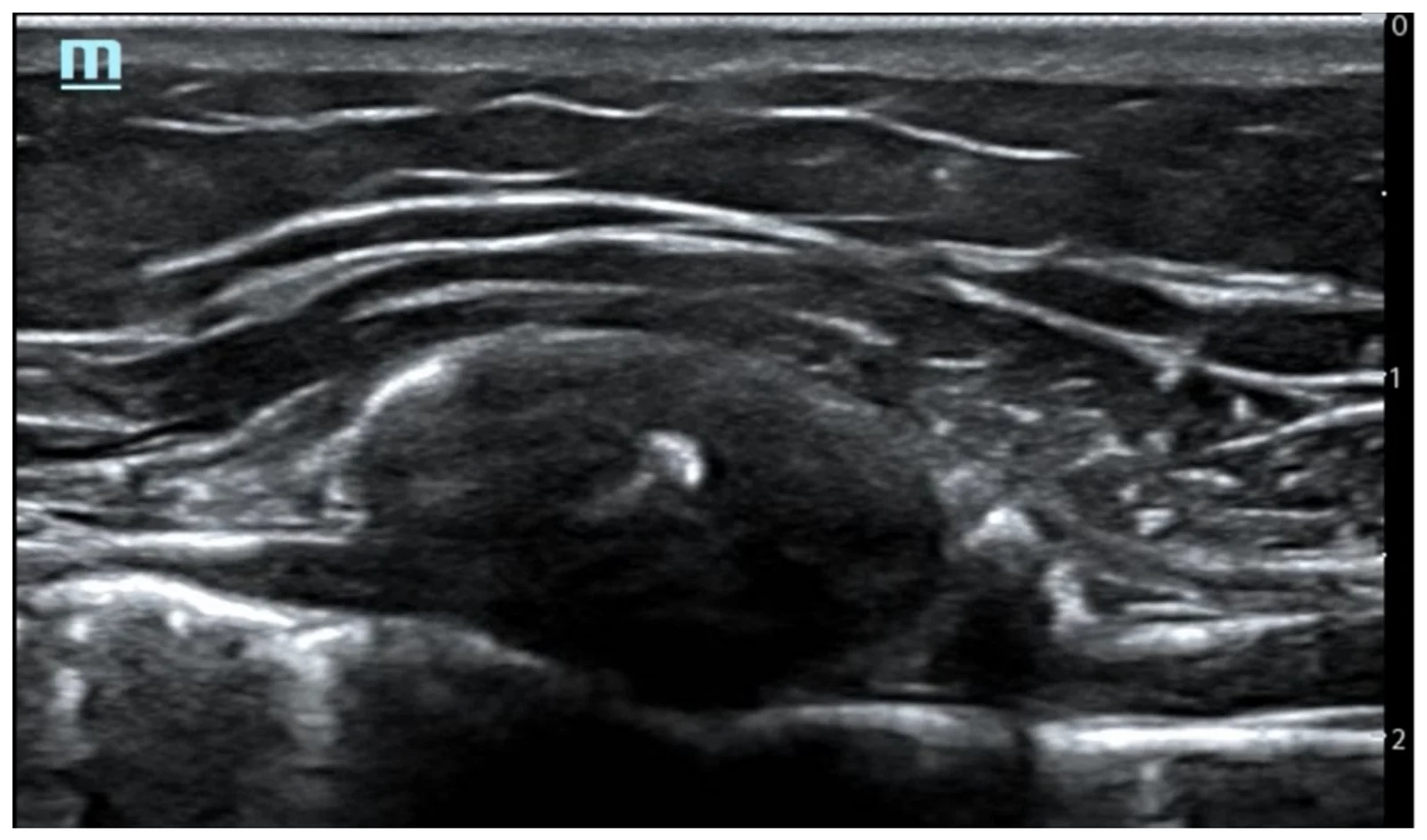
Ultrasound view of pectoralis major muscle resting on rib cartilage in parasternal line.

**Figure 4:**
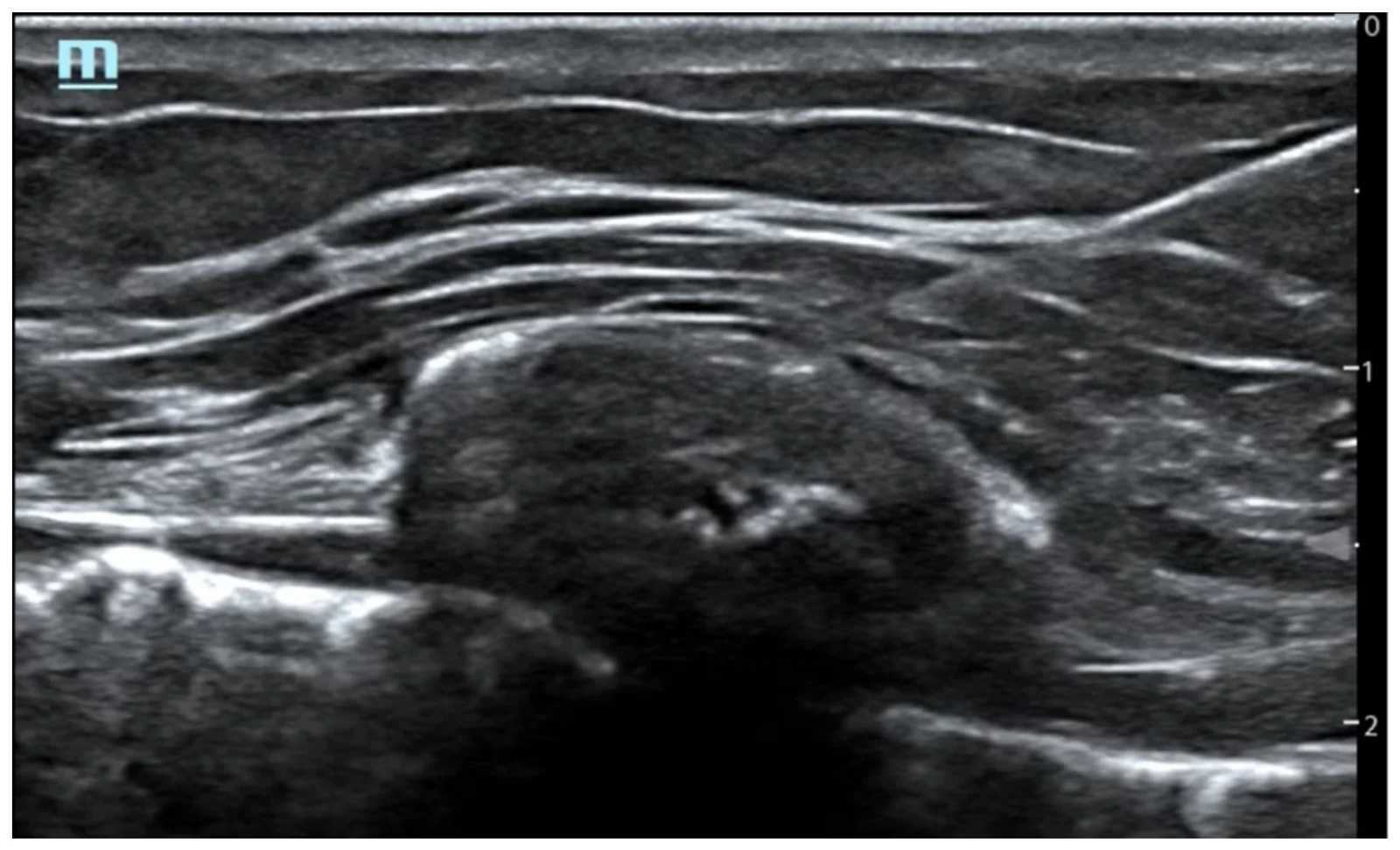
Ultrasound view of hydrodissection during modified SPIP block.

**Figure 5:**
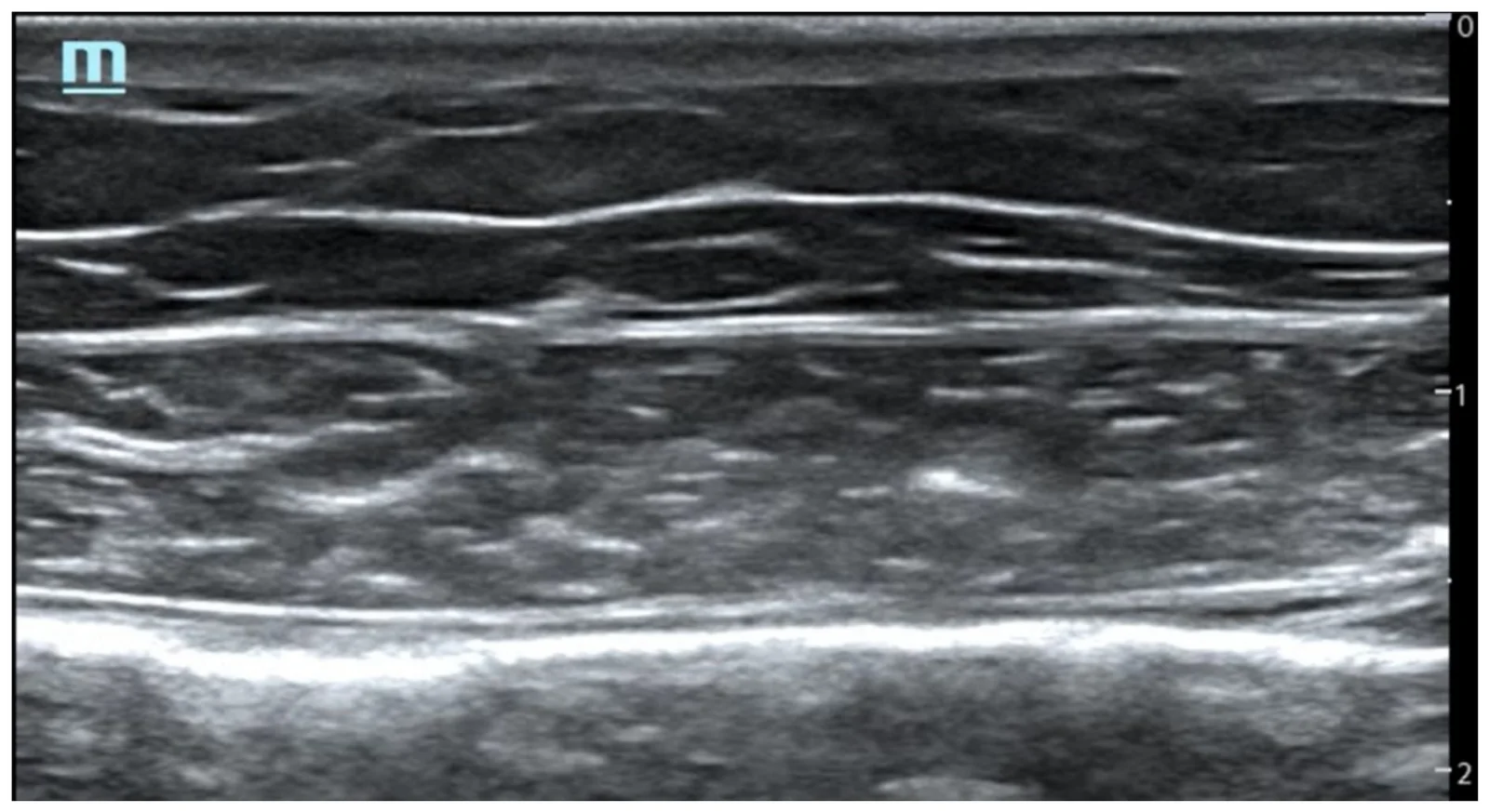
Ultrasound view of rectus abdominis muscle inside its sheath.

**Figure 6:**
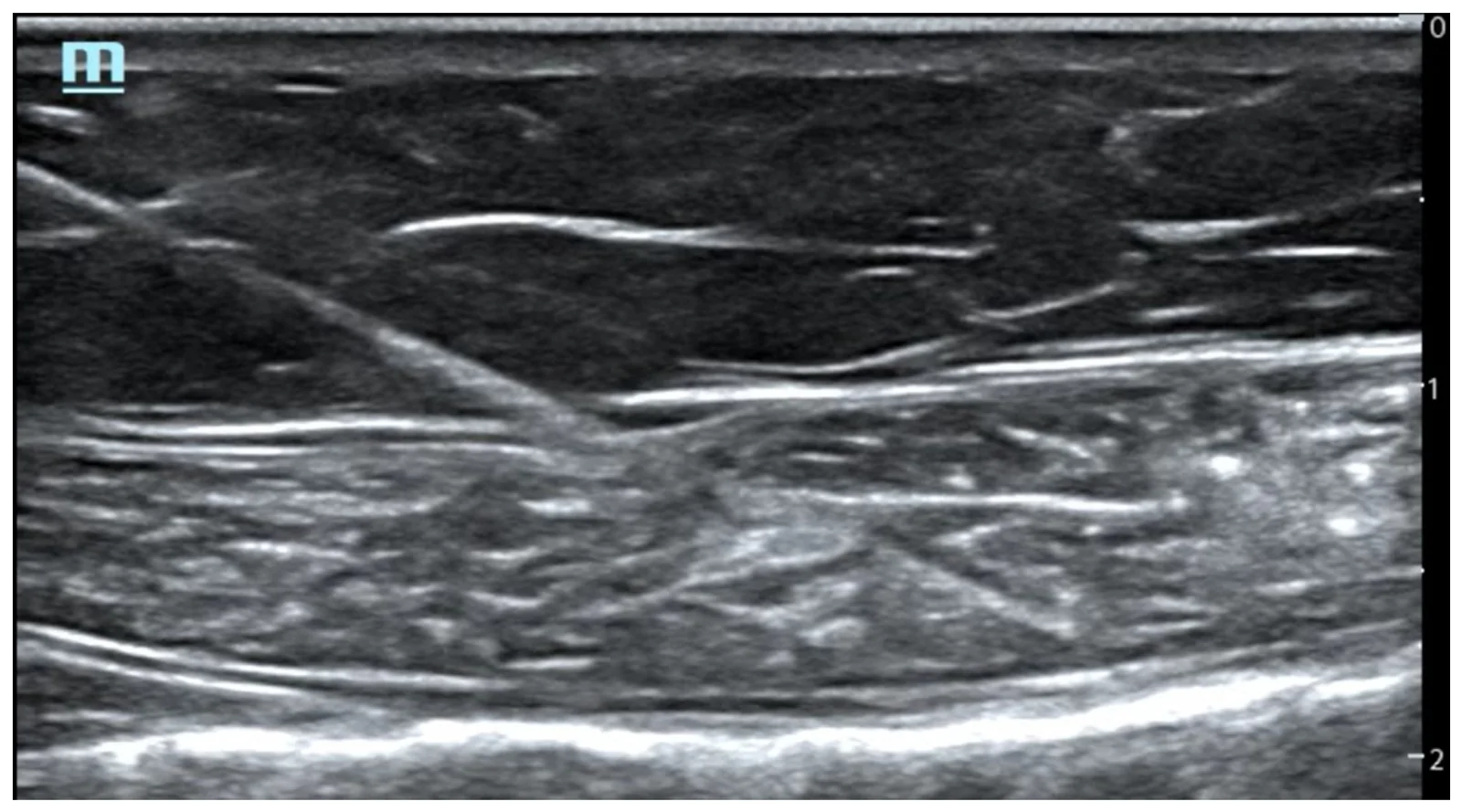
Ultrasound view of hydrodissection during RSB.

**Figure 7:**
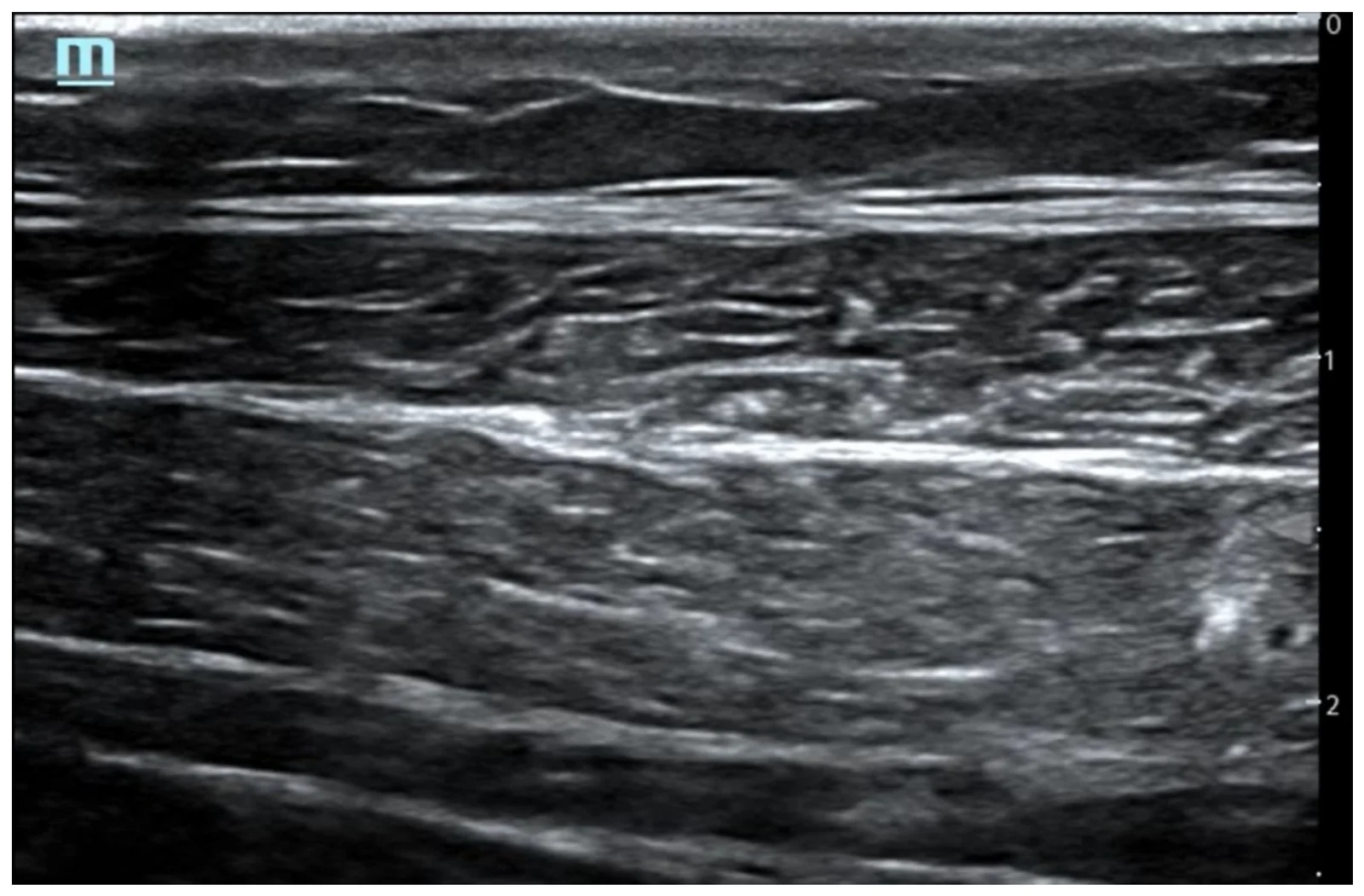
Ultrasound view of external oblique muscle, internal oblique muscle and transversus abdominis muscle in medial axillary line.

**Figure 8:**
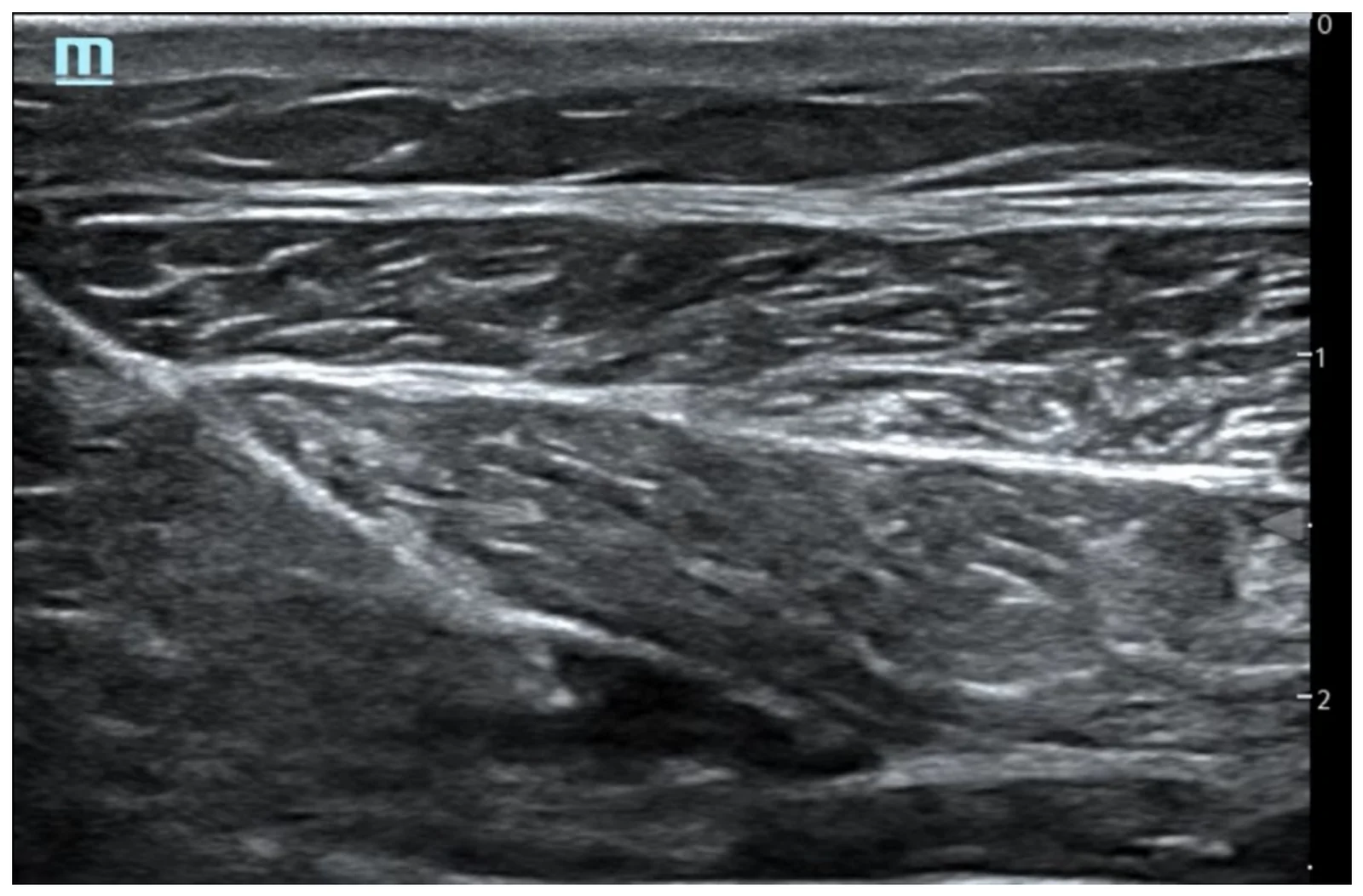
Ultrasound view of hydrodissection during TAPB.

**Table 1:** Description of the average pressure in the hydrodissection segment after baseline correction.

Block	n	Mean [PSI]	SD [PSI]	95% CI Mean [PSI]
**PECS I**	10	4.995	1.352	4.027 to 5.962
**SPIP block**	10	5.087	0.806	4.511 to 5.663
**RSB**	10	4.473	0.600	4.044 to 4.902
**TAPB**	10	5.503	0.525	5.127 to 5.878

**Table 2:** Parametric *t*-test for pairs: mean hydrodissection pressure after baseline correction.

Comparison	Mean Difference [PSI]	95% CI Lower [PSI]	95% CI Upper [PSI]	t	*p*	Holm-Adjusted *p*-Value	Cohen’s d Effect Size
**SPIP vs. PECS I**	0.0921	−0.6686	0.8529	0.2739	0.7903	0.7903	0.0866
**TAPB vs. RSB**	1.0293	0.3639	1.6947	3.4996	0.0067	0.0134	1.1067

**Table 3:** Parametric *t*-test for pairs: hydrodissection beta trend after baseline correction.

Comparison	Mean Difference [PSI/s]	95% CI Lower [PSI/s]	95% CI Upper [PSI/s]	t	*p*	Cohen’s d Effect Size
**SPIP vs. PECS I**	−0.005243	−0.021480	0.010994	−0.7304	0.4837	−0.2310
**TAPB vs. RSB**	0.001437	−0.008136	0.011010	0.3396	0.7420	0.1074

**Table 4:** Additional sensitivity analysis: permutation tests for mean hydrodissection pressure.

Comparison	Observed Difference [PSI]	Number of Permutations	Permutation *p*	Holm-Adjusted *p*-Value	Cohen’s d Effect Size
**SPIP vs. PECS I**	0.092	1024	0.809	0.809	0.087
**TAPB vs. RSB**	1.029	1024	0.010	0.020	1.107

**Table 5:** Pearson correlation coefficient between hydrodissection pressure and height, weight, and BMI of the cadavers.

Comparison	Cadaver Parameter	Pearson r	Adjusted *p*-Value
**PECS I-SPIP**	Height [cm]	−0.220	1.000
**PECS I-SPIP**	Weight [kg]	−0.149	1.000
**PECS I-SPIP**	BMI [kg/m^2^]	0.199	1.000
**RSB-TAPB**	Height [cm]	0.008	1.000
**RSB-TAPB**	Weight [kg]	0.200	1.000
**RSB-TAPB**	BMI [kg/m^2^]	0.533	0.675

## Data Availability

Data are included in the Supplementary Materials.

## Supplementary Materials

The following supporting information can be downloaded at: https://www.mdpi.com/article/10.3390/jcm15186979/s1, Figure S1: Q-Q plots of paired differences used to assess the normality assumption for the parametric paired analyses; Figure S2: Comparison of peaks P1–P4 detected within each block; Table S1: Cadaver descriptions; Table S2: Cadavers’s basic statistics; Table S3: Description of peaks P1–P4 within each block; Table S4: Results of the post hoc D1–D2 segment sensitivity analysis; Supplementary Methods: Detailed automated detection of characteristic points in pressure–time curves.
