# Supplementary material for: Continuous Needle Tip Injection Pressure During Hydrodissection in Four Fascial Plane Blocks: A Fresh Human Cadaver Study
Source: J Clin Med. 2026 Sep 9;15(18):6979. doi: 10.3390/jcm15186979 (PMC13607384; doi:10.3390/jcm15186979)
Supplement: Supplementary file 1 [file jcm-15-06979-s001.zip › Supplementary Materials.pdf]

Section S1

Supplementary Data

Supplementary Table S1. Cadaver descriptions.

Cadaver ID	Sex	Height [cm]	Weight [kg]	BMI [kg/m^2]
**1**	Female	162,000	55,000	20,957
**2**	Female	158,000	53,000	21,231
**3**	Male	177,000	72,000	22,982
**4**	Male	175,000	70,000	22,857
**5**	Male	170,000	64,000	22,145
**6**	Female	160,000	60,000	23,438
**7**	Male	172,000	65,000	21,971
**8**	Male	180,000	75,000	23,148
**9**	Female	155,000	57,000	23,725
**10**	Female	163,000	62,000	23,335

Supplementary Table S2. Cadavers's basic statistics.

Characteristic	n	Mean	SD	95% CI low	95% CI high
**Height [cm]**	10	167,200	8,702	160,975	173,425
**Weight [kg]**	10	63,300	7,364	58,032	68,568
**BMI [kg/m^2]**	10	22,579	0,954	21,896	23,262

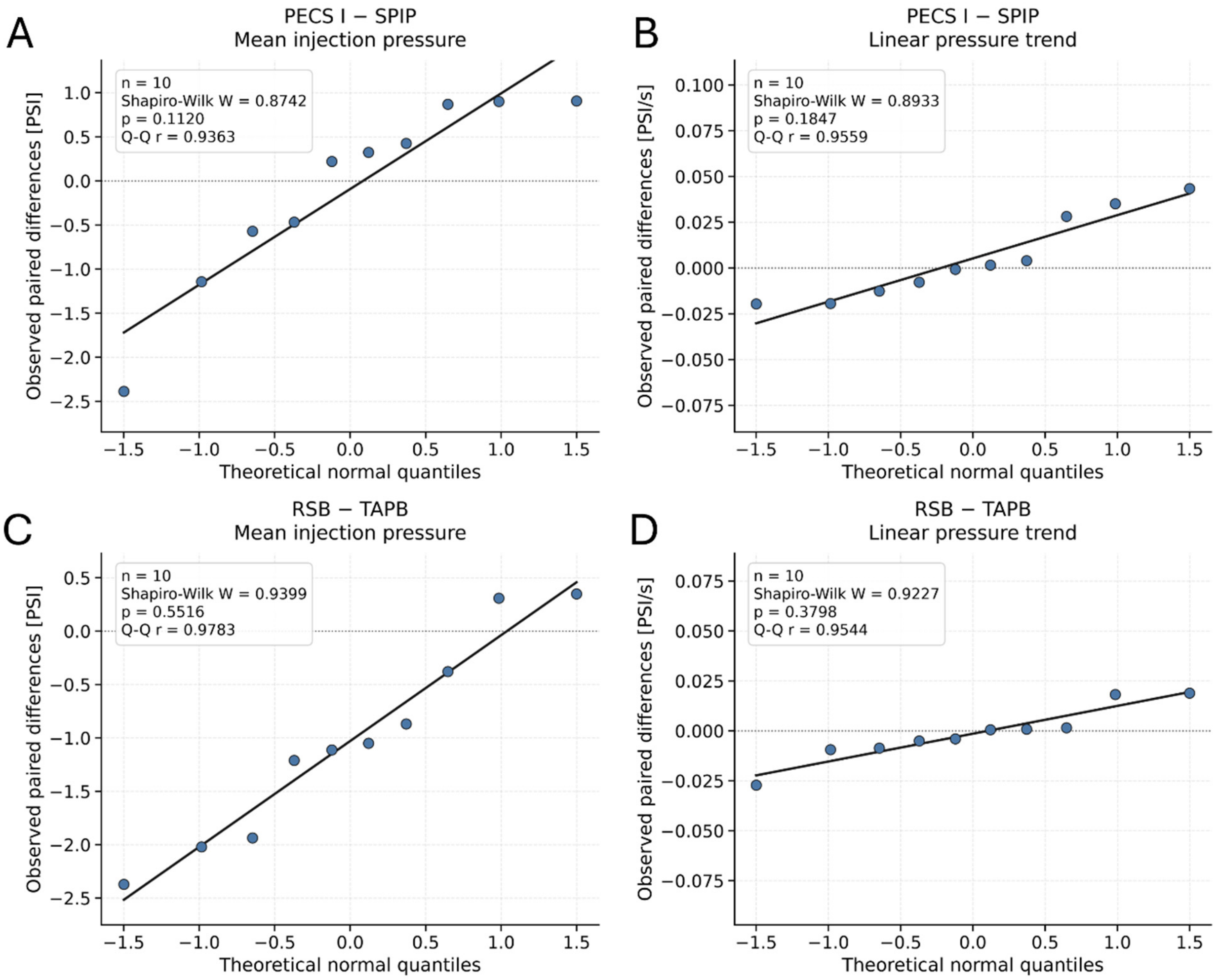

Supplementary Figure S1. Q-Q plots of paired differences used to assess the normality assumption for the parametric paired analyses. Panels show the distribution of within-specimen differences for mean baseline-corrected injection pressure during stable hydrodissection and for the corresponding linear pressure trend. A) Mean injection pressure, PECS I block versus superficial parasternal block; B) linear pressure trend, PECS I block versus SPIP; C) mean injection pressure, RSB versus TAPB; D) linear pressure trend, RSB versus TAPB. The points represent individual cadaveric specimens.

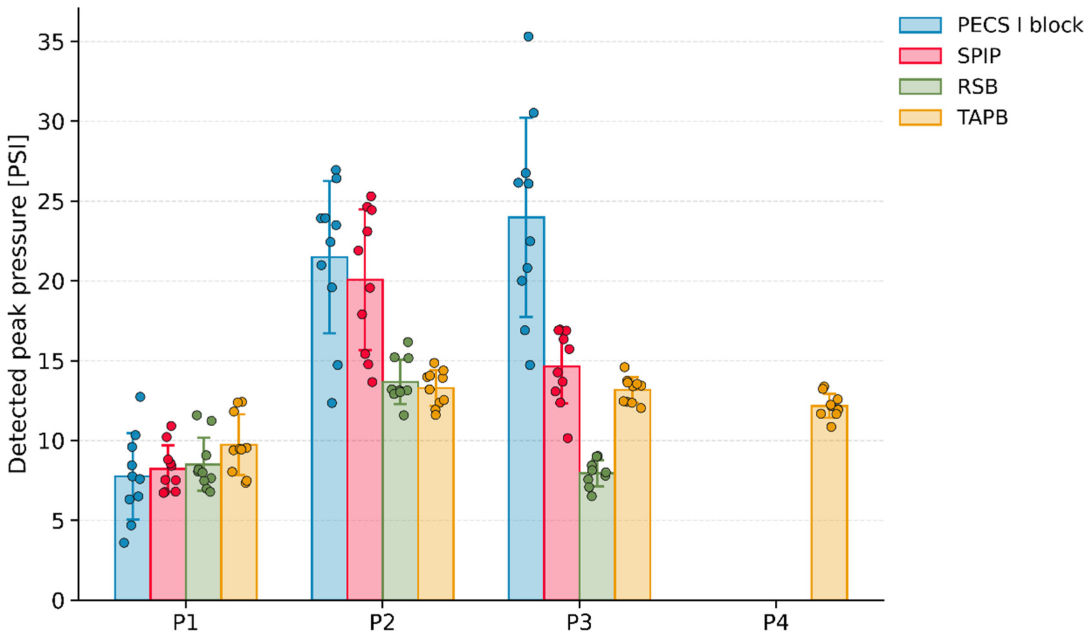

Supplementary Figure S2. Comparison of peaks P1–P4 detected within each block.

Supplementary Table S3. Description of peaks P1–P4 within each block.

Block	P1 [PSI]	P2 [PSI]	P3 [PSI]	P4 [PSI]
**PECS I**	7.758 ± 2.704	21.485 ± 4.762	23.979 ± 6.251	NA
**Parasternal**	8.229 ± 1.456	20.077 ± 4.417	14.640 ± 2.318	NA
**RSB**	8.503 ± 1.661	13.669 ± 1.386	7.951 ± 0.833	NA
**TAPB**	9.737 ± 1.901	13.285 ± 1.119	13.166 ± 0.806	12.170 ± 0.753

Supplementary Table S4. Results of the post hoc D1–D2 segment sensitivity analysis.

D shift [s]	SPIP–PECS I mean diff [psi]	SPIP–PECS I Holm-adjusted p-value	SPIP–PECS I exact p	TAPB–RSB mean diff [psi]	TAPB–RSB Holm-adjusted p-value	TAPB–RSB exact p
**-5**	0.150	0.6933	0.6973	1.085	0.0185	0.0137
**-4**	0.198	0.6032	0.6230	1.087	0.0149	0.0137
**-3**	0.178	0.6209	0.6484	1.069	0.0141	0.0117
**-2**	0.134	0.6970	0.7031	1.036	0.0157	0.0137
**-1**	0.101	0.7700	0.7910	1.022	0.0150	0.0137
**0**	0.092	0.7903	0.8086	1.029	0.0134	0.0098
**1**	0.085	0.8057	0.8242	1.034	0.0124	0.0098
**2**	0.077	0.8241	0.8379	1.038	0.0115	0.0098
**3**	0.069	0.8444	0.8574	1.042	0.0107	0.0098
**4**	0.058	0.8681	0.8789	1.044	0.0101	0.0098
**5**	0.047	0.8947	0.9023	1.047	0.0096	0.0098

Section S2

Supplementary Methods. Detailed automated detection of characteristic points in pressure–time curves

The following implementation-level details supplement the abbreviated procedure described in the main Methods. The segmentation procedure was developed using the same recordings included in the present study. The numerical settings described below were established empirically during iterative visual development to provide an operational segmentation of characteristic signal transitions. They were not intended to represent physiological or clinical thresholds.

Pressure–time recordings were analyzed using a custom deterministic signal-processing algorithm implemented in Python. Pressure was analyzed in PSI (pounds per square inch). The time axis was referenced to the first valid timestamp in each recording. Recordings were nominally sampled at approximately 100Hz. The pressure signal was sequentially smoothed using a centered 11-sample median filter, corresponding to approximately 0.11s, and an exponentially weighted moving average with a span parameter of 31 samples ($\alpha = 0.0625$, where α denotes the EWMA smoothing coefficient). All characteristic points and their corresponding pressure values were determined from the filtered signal. The reported parameter values were held constant during batch processing, except for the block-specific number of expected pressure peaks.

The 5-s delay before the W search served as a procedural lead-in period before the expected onset of needle insertion. The 0.77psi and 27.5% criteria were used to distinguish the initial sustained pressure rise from smaller fluctuations, whereas the 35% criterion was used to retain relevant high-pressure events for delimiting D1. The asymmetric 0.15psi and 0.30psi plateau tolerances allowed limited downward variation while restricting backward extension of D1 into the preceding rising portion of the signal.

A low-variability reference interval was identified as the longest continuous segment of the filtered signal satisfying (1):

$$\max(p) - \min(p) \leq T, \quad (1)$$

where:

p denotes the filtered pressure values within the candidate interval, expressed in psi;

$\max(p)$ and $\min(p)$ denote the maximum and minimum filtered pressure values within that interval, respectively;

T denotes the maximum allowable total pressure range within the interval, expressed in psi.

The threshold T was calculated as follows (2):

$$T = \max[0.009 \text{ psi}, \ 0.08(Q_{0.95} - Q_{0.05})], \quad (2)$$

where:

T denotes the maximum allowable total pressure range, expressed in psi;

$Q_{0.95}$ and $Q_{0.05}$ denote the 95th and 5th percentiles of the filtered pressure signal, respectively;

0.009psi denotes the minimum absolute range threshold;

0.08 denotes the dimensionless relative range factor;

max[·] denotes selection of the greater of the two threshold values.

The interval was required to contain at least 200 consecutive samples, corresponding to approximately 2s. In all analysed recordings, the identified low-variability interval occurred after the puncture-related phase and was therefore interpreted as the stable post-puncture pressure phase.

Point W represented the algorithmically detected initial sustained pressure rise associated with needle insertion. The search began 5s after the start of the recording and ended at the beginning of the low-variability interval. For each candidate sample i, the forward pressure increase was calculated over 60 samples, corresponding to approximately 0.60s (3):

$$\Delta p_i = p_{i+60} - p_i, \quad (3)$$

where:

Δp_i denotes the forward pressure increase calculated for candidate sample i, expressed in psi;

p_i denotes the filtered pressure at candidate sample i, expressed in psi;

p_{i+60} denotes the filtered pressure 60 samples after candidate sample i, expressed in psi;

i denotes the index of the candidate sample;

60 denotes the forward offset in samples, corresponding to approximately 0.60s at an effective sampling rate of approximately 100Hz.

Point W was defined as the first sample for which Δp_i exceeded both an absolute threshold of 0.77psi and a recording-specific threshold corresponding to 27.5% of the largest forward pressure increase within the analysed interval. If no sample fulfilled both criteria, the sample associated with the largest positive forward pressure increase was retained, and application of this fallback rule was recorded in the algorithm output.

Point P1 was defined as the first prominent pressure maximum following W. The search was restricted to the first 30s of the recording. After the signal exceeded the pressure at W by at least 2.0psi, the algorithm tracked the highest subsequently observed pressure. The candidate maximum was accepted as P1 when it was followed by at least eight consecutive samples, corresponding to approximately 0.08s, with pressure values at least 0.20psi below the candidate maximum. If this decrease criterion was not fulfilled before the end of the search interval, the highest value reached during the initial pressure increase was retained as P1.

Point D1 was defined as the algorithmically estimated onset of the stable post-puncture pressure plateau. Because identifying P2–P4 required D1 to delimit the analysis interval, D1 was initially estimated using a preliminary set of high-pressure points. Preliminary P1 was identified using the procedure described above. Additional candidates occurring after W and within the first 30s of the recording were ranked according to pressure amplitude. Candidates were required to exceed both the pressure at W by at least 1.0psi and 35% of the maximum pressure increase within this interval. Up to four preliminary points separated by at least 80 samples, corresponding to approximately 0.80s, were retained and ordered chronologically. These preliminary points were used exclusively to delimit the search for the segment D1-D2 and were not necessarily identical to the subsequently identified P2–P4 points.

The lowest filtered pressure value occurring after the last preliminary point and before the beginning of the low-variability interval was used as the initial drop candidate. This candidate was shifted 5s forward to reduce the influence of the immediate post-peak transient. The algorithm then moved backwards from the shifted position to identify the earliest sample consistent with the local pressure plateau. Plateau consistency was evaluated relative to the median filtered pressure within the subsequent 1.5-s window. A sample was considered consistent with the plateau when its pressure did not exceed the local median by more than 0.15psi and was not more than 0.30psi below it. The backward search was terminated after five consecutive samples failed these criteria, and the earliest preceding sample satisfying the plateau criteria was assigned as D1.

Final pressure maxima P2–P4, where applicable, were identified between P1 and D1. Candidate points were required to constitute local maxima, exceed the pressure at W by at least 1.0psi and reach at least 35% of the maximum pressure increase observed between P1 and D1. For each candidate, the differences between the candidate maximum and the minimum pressure within 1.2-s windows on its left and right sides were calculated. Both differences were required to be at least 0.45psi. Among the candidates satisfying these criteria, the highest maxima occurring at least 1s after P1 and separated from one another by at least 1s were retained. The selected points were ordered chronologically and labelled P2, P3 and, where applicable, P4. The expected total number of peaks was specified before processing according to the block type and the anticipated number of tissue interfaces traversed along the planned needle trajectory. The block-specific expected peak count was used only for final descriptive P1–P4 labeling and did not determine W or the D1–D2 segment.

Point D2 was defined exactly 40s after D1. When no sample was recorded at the exact D2 time, the corresponding pressure value was calculated by linear interpolation between the adjacent valid samples. D2 was not assigned when the target time was outside the available recording period.

The final P1–P4 labels were used only for descriptive summaries within each block and were not used in the formal between-technique comparisons.
